# Estimated specific antibody-based true sero-prevalences of canine filariosis in dogs in Central Europe and the UK

**DOI:** 10.1007/s00436-022-07695-1

**Published:** 2022-10-19

**Authors:** Jeannine E. Fehr, Manuela Schnyder, Deborah E. Joekel, Nikola Pantchev, Mindaugas Sarkunas, Paul Torgerson, Peter Deplazes

**Affiliations:** 1 Institute of Parasitology, Vetsuisse Faculty, University of Zurich, Winterthurerstrasse 266a, 8057 Zurich, Switzerland; 2 IDEXX Laboratories, 70806 Kornwestheim, Germany; 3 Lithuanian University of Health Sciences, Veterinary Academy, Tilzes str. 18, 47181 Kaunas, Lithuania; 4 Vetsuisse Faculty, Section of Epidemiology, University of Zurich, Winterthurerstrasse 270, 8057 Zurich, Switzerland

**Keywords:** Filarial-specific antibodies, True prevalence, Dogs, *Dirofilaria repens*, Austria, Denmark, Germany, Lithuania, Poland, Switzerland, UK

## Abstract

Dirofilariosis is a vector-borne disease mainly caused by *Dirofilaria immitis* and *Dirofilaria repens*. In contrast to the known endemicity of dirofilariosis in southern and south-eastern Europe, information on the distribution of *D. repens* in Central-Europe is fragmentary. We tested 8877 serum samples from dogs from Austria, Denmark, Germany, Italy, Lithuania, Poland, Switzerland and the UK using an ELISA detecting filarial-specific antibodies, hypothesising higher occurrence of *D. repens*. Based on two overlapping frequency distributions, presumed negative samples had a mean optical density (OD) value of 0.097, representing 97.45% of all samples. Presumed positive samples, representing 2.55% of all sera, had a mean OD value of 0.287. Test prevalence based on the calculated cut-off was 3.51% for all sera (4.36% for Austria, 1.94% for Denmark, 1.39% for Germany, 3.37% for Italy, 6.90% for Lithuania, 6.99% for Poland, 0.77% for Switzerland and 0.0% for the UK, respectively). The bimodal distribution, representing overlapping distributions of OD values from positive and negative dogs, enabled the assignment of a probability of true infection status to each dog. Mean probabilities of true infection status across groups, based on the postal codes of origin, allowed us to estimate and map true prevalences. For all countries, except the UK, the true prevalence was lower than the test prevalence. The large number of serum samples and the use of a non-gold standard analytical method allowed us to create a more realistic picture of the distribution of *D. repens* in Central Europe and the UK.

## Introduction

In Europe, dogs and wild carnivores are hosts of a variety of filarial species. Canine filariae belong to the family of the Onchocercidae, whereof the clinically relevant representatives are *Dirofilaria immitis* and *D. repens*, whilst *Acanthocheilonema reconditum*, *Dipetalonema* (*A.*) *dracunculoides* and *Cercopithifilaria* spp. are mostly apathogenic (Ionică et al. 2015; Sonnberger et al. 2021). Mosquito species of the genera *Culex*, *Aedes* and *Anopheles* serve as vectors and intermediate hosts, transmitting infectious *Dirofilaria* third-stage larvae to a susceptible host (Cancrini et al. 2007; Morchon et al. 2012; Silaghi et al. 2017). *Dirofilaria immitis* causes ‘heartworm disease’ in dogs and occasionally cats and ferrets; *D. repens* is at the origin of subcutaneous/ocular ‘skinworm’ disease (Genchi et al. 2011). Both *Dirofilaria* spp. can cause subcutaneous, ocular and pulmonary dirofilariosis in humans which act as dead-end hosts (Pampiglione and Rivasi 2000; Simon et al. 2012).

*Dirofilaria immitis* and *D. repens* predominantly occur in southern and south-eastern European areas, respectively. For *D. immitis*, a trend to spread in north-eastern Europe has been observed in recent decades (Genchi et al. 2011). The northern border of transmission of *D. immitis* is not well documented to date, but cases have been identified as north as Central France, Southern Switzerland, Northern Italy, Slovakia, Bulgaria, Hungary and Ukraine (Deplazes et al. 1995; Farkas et al. 2020; Hermosilla et al. 2006; Laidoudi et al. 2019; Mendoza-Roldan et al. 2020; Panayotova-Pencheva et al. 2020; Petruschke et al. 2001; Sassnau et al. 2014a; Széll et al. 2020).

The endemic areas of *D. repens* mostly overlap with those of *D. immitis* but also extend significantly northwards. In particular, in the north-eastern parts of Central Europe, including Poland, Lithuania, Ukraine and the western and south-western as well as eastern parts of Russia, this species has become an important zoonotic disease in recent decades, causing thousands of infections in humans (Kondrashin et al. 2020; Rossi et al. 2015). For instance, in Ukraine *D. repens* was confirmed in 1′465 human cases between the years 1996 and 2012, based on a Europe-wide unique nationwide mandatory reporting for human cases since 1975 (Salamatin et al. 2013). Surveillance of human subcutaneous and ocular dirofilariosis in the Russian Federation and Belarus revealed overall 1′272 cases between 1997 and 2013, mainly from south-western regions of the Russian Federation, but with a northwards spread (Kartashev et al. 2015; Moskvina and Ermolenko 2018). Data collected between 1981 and 2011 from the Russian Federation, Ukraine, Belarus and Kazakhstan were applied to a climatic prediction model and regional warming was found to increase the annual generations of *Dirofilaria* spp. and thus increase suitable transmission areas by 18.5% in 2030 (Kartashev et al. 2014). Previous autochthonous cases of *D. repens* in humans have been described in Poland (Cielecka et al. 2012), Italy (Pampiglione et al. 2001), nine countries of the Balkan Peninsula (Tasić-Otašević et al. 2015), Hungary (Dóczi et al. 2015) and Slovakia (Babal et al. 2008; Boldiš et al. 2020). More recently, between 2017 and 2022, single cases of human *D. repens* infections were reported again from the Balkan area, in Serbia (Krstic et al. 2017), Croatia (Skrinjar et al. 2022), Bulgaria (Velev et al. 2019) and Romania (Ciuca et al. 2018), and further cases from Greece (Bozidis et al. 2021) and Italy (Ahmed et al. 2022), Interestingly, also in Austria, overall 39 human cases of dirofilariosis were identified between 1978 and 2020, with a general increasing course since 1998 (Riebenbauer et al. 2021). In addition, single human cases were reported from south-eastern France (Hennocq et al. 2020) and Germany (Uslu et al. 2017), confirming a north- and westwards trend in Europe.

*Dirofilaria repens* infections in dogs are also well documented in north-eastern, northern and southern Europe (Alsarraf et al. 2021; Genchi et al. 2011; Pantchev et al. 2009; Tarello 2011). However, in Central Europe, the epidemiological situation of *D. repens* north of the Alps is not fully known and only fragmentary data are available. For decades, in most cases of *D. repens* infections diagnosed in dogs in Central Europe and Great Britain the dogs originated from classical endemic areas or had a travel history with exposure in these areas. In Austria, for instance, most of previously detected cases in dogs were suspected to be imported. However, single cases detected in humans and dogs indicated autochthonous transmission of *D. repens* in eastern Austria (Fuehrer et al. 2016). Interestingly, in a surveillance programme that included 7632 mosquitoes, DNA from *D. repens* was amplified in two of 437 pools: the positive pools were from eastern Austria (Silbermayr et al. 2014), close to Hungary. Accordingly, the almost triplication of documented cases between 2014 and 2017 suggested endemic establishment in the country (Sonnberger et al. 2020). Also of interest is the so-called ´stable´ transmission of *D. repens* in north-eastern Germany and positive cases in dogs in the same region (Czajka et al. 2014; Sassnau et al. 2013). On the other side, *D. repens* was shown to be the most frequently imported filarial infection in Germany already in 2008–2010: in more than 8000 dogs with travel history or imported from endemic countries, *D. repens* was identified in 42 dogs, with Slovenia and Hungary being the most frequent mentioned countries (Pantchev et al. 2011). In fact, the endemic situation in these and surrounding countries such as the Czech Republik and Slovakia is confirmed by recent studies (Farkas et al. 2020; Jurankova et al. 2022; Martina et al. 2021). Furthermore, increasing reported cases of canine dirofilariosis due to *D. repens* in Lithuania, Latvia, Poland and Belarus confirm the trend for spreading northeastern (Alsarraf et al. 2021; Sabūnas et al. 2019).

Suspected reasons for the increasing number of reports of *D. repens* in northern Europe are the organised import of infected dogs from animal shelters protecting stray and unwanted dogs in the endemic countries, as well as the concurrent changing travel habits of humans taking their pets more frequently on trips. Furthermore, global trade can lead to the spread of infective vectors, allowing them to infect susceptible hosts in non-endemic regions (Genchi et al. 2011).

However, for instance, although predicted, the establishment of *D. repens* in the southern parts of Switzerland in areas where *D. repens* has been found several times in individual dogs has not been progressed in the last 30 years (Fuehrer et al. 2021). Therefore, some further unknown epidemiological factors may be associated with transmission of the parasite.

Diagnosis of filarial infections in dogs is achieved by concentration of blood microfilariae using the Knott Test (Knott 1939) or a filter method (Bell 1967). Morphometric measurements of microfilariae fixed with the Knott Test allow the differentiation of *D. immitis* and *D. repens* from the other smaller filarial species (Magnis et al. 2013). Another, older approach is the use of acid phosphatase staining with isolated microfilariae for morphological differentiation (Chalifoux and Hunt 1971; Peribáñez et al. 2001). Today, genetic identification of individual microfilariae at species level can easily be achieved by PCR (Rishniw et al. 2006). However, all microfilaria-related diagnostic methods lack high sensitivity due to long lasting prepatent infections, intermittent microfilaremia due to microfilarial periodicity, same-sex infections, anthelmintic-induced adult sterility and infections in which microfilariae have been destroyed by anthelmintics or by an immune response (Bowman and Mannella 2011; Rawlings et al. 1982).

For the identification of heartworm infections, detection of *D. immitis* antigen produced by adult females in the definitive host’s blood is the most common diagnostic procedure today (Weil 1987). There are commercially available ELISA kits that detect infections with at least one female worm and are therefore described as highly sensitive and nearly 100% specific (Atkins 2003; Lee et al. 2011). However, cross-reactions with sera from dogs infected with *Angiostrongylus vasorum* have been documented in 3/6 commercially available test kits (Schnyder and Deplazes 2012). Moreover, it has been suggested that the use of slow-kill heartworm treatments can induce immune complexes that lead to misleading false-negative results (Drake et al. 2015). If those complexes are destroyed by heat treatment, samples convert from negative to positive, as it has been shown for 7% of samples in a study from the USA (Velasquez et al. 2014).

Serology for the detection of specific antibodies has previously been considered to have low specificity, because of cross-reactivity with many other non-filarial nematodes (Grieve et al. 1981; Grieve and Knight 1985; Sisson et al. 1985). Recently, a monoclonal antibody based on-plate affinity purification of a crude *D. immitis* antigen was shown to not cross-react with sera from dogs experimentally infected with non-filarial nematodes such as *Angiostrongylus vasorum*, *Toxocara canis*, *Ancylostoma caninum* and *Trichuris vulpis* (Joekel et al. 2017). Furthermore, few positive reactions were found in dogs with documented natural infections with *Crenosoma vulpis, A. vasorum* and *Capillaria aerophila* (syn. *Eucoleus aerophilus*), but in these dogs previous exposure to filarial species could not be excluded. On the other hand, more than 50% of the dogs with low-pathogenic filarial infections with *Acanthocheilonema*, *Dipetalonema* spp. (Joekel et al. 2017) and *Cercopithifilaria* spp. (Deplazes, personal communication) had positive antibody reactions. Therefore, this test was defined as filarial-specific with a sensitivity of 93.8% for *D. immitis* patent infections and 100% for *D. repens* patent infections. Seroconversion of dogs experimentally infected with *D. repens* occurred between 24- and 80-day post inoculation (dpi) with third-stage larvae, much earlier than the beginning of patency (161–238 dpi) (Petry et al. 2015). Due to these test characteristics, the presented ELISA represents a suitable epidemiological tool, especially for the study of *Dirofilaria* spp. in low or non-endemic areas, where transmission of the apathogenic species is scarce (Joekel et al. 2017). To date, there have been no large-scale studies with high numbers of tested dogs outside Southern Europe. The aim of this work was to determine the distribution patterns of canine filarial infections in dogs with overall 8877 blood samples from Austria, Denmark, Germany, Italy, Lithuania, Poland, Switzerland and the UK.

## Material and methods

### Dog blood samples

The samples from Austria, Denmark, Germany, Italy, Poland and the UK were previously collected as part of other studies (Guardone et al. 2013; Schnyder et al. 2013a, 2013b). The samples from Italy were used as a positive known endemic area for a variety of filarial species (Cringoli et al. 2001; Magi et al. 2008, 2012; Otranto et al. 2013; Traversa et al. 2010). The samples from Lithuania originated from Kaunas (Central Lithuania; pet and sheltered dogs) and Klaipeda (Western Lithuania; pet dogs). The samples from Switzerland were collected for a sero-epidemiological survey on *Angiostrongylus vasorum* (Lurati et al. 2015). All sera were collected from dogs presented at veterinary clinics for different reasons and were complemented by corresponding data on the owner’s postal code. Due to data protection, no further information about the animal or the animal owner was available.

### ELISA for detection of filarial-specific antibodies

The ELISA was performed as previously described (Joekel et al. 2017), with following modifications: a large batch of monoclonal antibodies (mAb Di36/1) was prepared. All ELISA plates included three positive control sera from dogs with experimental *D. repens* infections (Joekel et al. 2017), two negative controls from healthy dogs to adapt the plate-to-plate variation, a background and a conjugate control. The cut-off value was calculated for each country as follows: mean plus three standard deviations of the ELISA optical density (OD) values (measured at 405 nm) of sera from 300 dogs per country (*n* = 228 for Denmark, year of sampling: 2017). As an additional procedure, all sera that resulted positive (above the cut-off value calculated for each country) were retested in an ELISA without *D. immitis* somatic antigen (defined as control ELISA) to exclude false-positive reactions between the murine monoclonal antibodies and the dog sera. For the positive sera in the control ELISA, we included a restriction criterion: if the OD value of the retested sample without antigen was higher than the value of the test ELISA with somatic antigen, the sample was considered uninterpretable and excluded from further analyses. Furthermore, 53 samples from Lithuania were re-examined by Knott test and in each positive sample (*n* = 8) 10 microfilariae were measured morphometrically (Magnis et al. 2013). Eventually, in 3 cases the diagnosis was confirmed by PCR (Cafarelli et al. 2019).

### Statistical and data analysis

The frequency distribution of the OD values was assumed to be a mixture distribution with two or more modes. The probability distribution with the highest mode represents the OD values of the assumed positive dogs and the probability distribution(s) of the lower mode(s) represents the assumed negative dogs. As a first stage mixture distribution was analysed as a mixture of normal distributions using the R package mclust (Scrucca et al. 2016). This optimised the number of mixture distributions using BIC. As a second step the number of distributions was further reduced by combining mixture components for clustering (Baudry et al. 2010). This resulted in the most likely probability distributions to which negative and positive dogs belonged and thus enabled a probability of true infection status to be assigned to each dog. Mean probabilities of the true infection status across groups of dogs represent an estimate of true prevalence.

### Mapping, geographical distribution

Based on the postal codes of the addresses of origin of the samples, the mean probabilities were mapped into 1st or 2nd level administrative districts. First or second level was chosen so that districts of similar area could be readily compared across different countries. Mean prevalences were calculated when there were 5 or more samples available in a district. Districts with fewer than 5 samples were excluded to avoid bias created by the chance finding of one high OD value in a very small sample size. The mean prevalences were plotted into each district using 1st or 2nd administrative level shape files using R library ggplot2 (Wickham 2016).

## Results

Table 1 shows the total number of tested dog samples for each country, the number of sera above the cut-off OD values, the calculated prevalence of each country and the corresponding true prevalence.

**Table 1:** Central European sero-epidemiological study for the detection of filarial-specific antibodies in dogs

Country, year of sampling	Number of included and excluded (in brackets)^a^ tested dog sera	Above cut-off	Calculated test prevalence, in %	True prevalence, in %
Austria, 2015	550 (0)	24	4.36	3.00
Denmark, 2012 (*n* = 1216) and 2017 (n = 228)	1440 (4)	28	1.94	1.48
Germany, 2010	1586 (9)	22	1.39	0.97
Italy, 2017	771 (2)	26	3.37	3.04
Lithuania, 2017	232 (0)	16	6.90	5.85
Poland, 2011	2716 (12)	190	6.99	4.62
Switzerland, 2012	521 (2)	4	0.77	0.58
UK, 2010	1028 (4)	0	0.00	0.44
All countries	8844 (33)	310	3.51	2.55

^a^Number of samples excluded from analysis due to a false-positive reaction resulting from a negative delta in the subtraction of the OD value from the specific reaction minus the unspecific reaction without the specific antigen

**Fig. 1:**
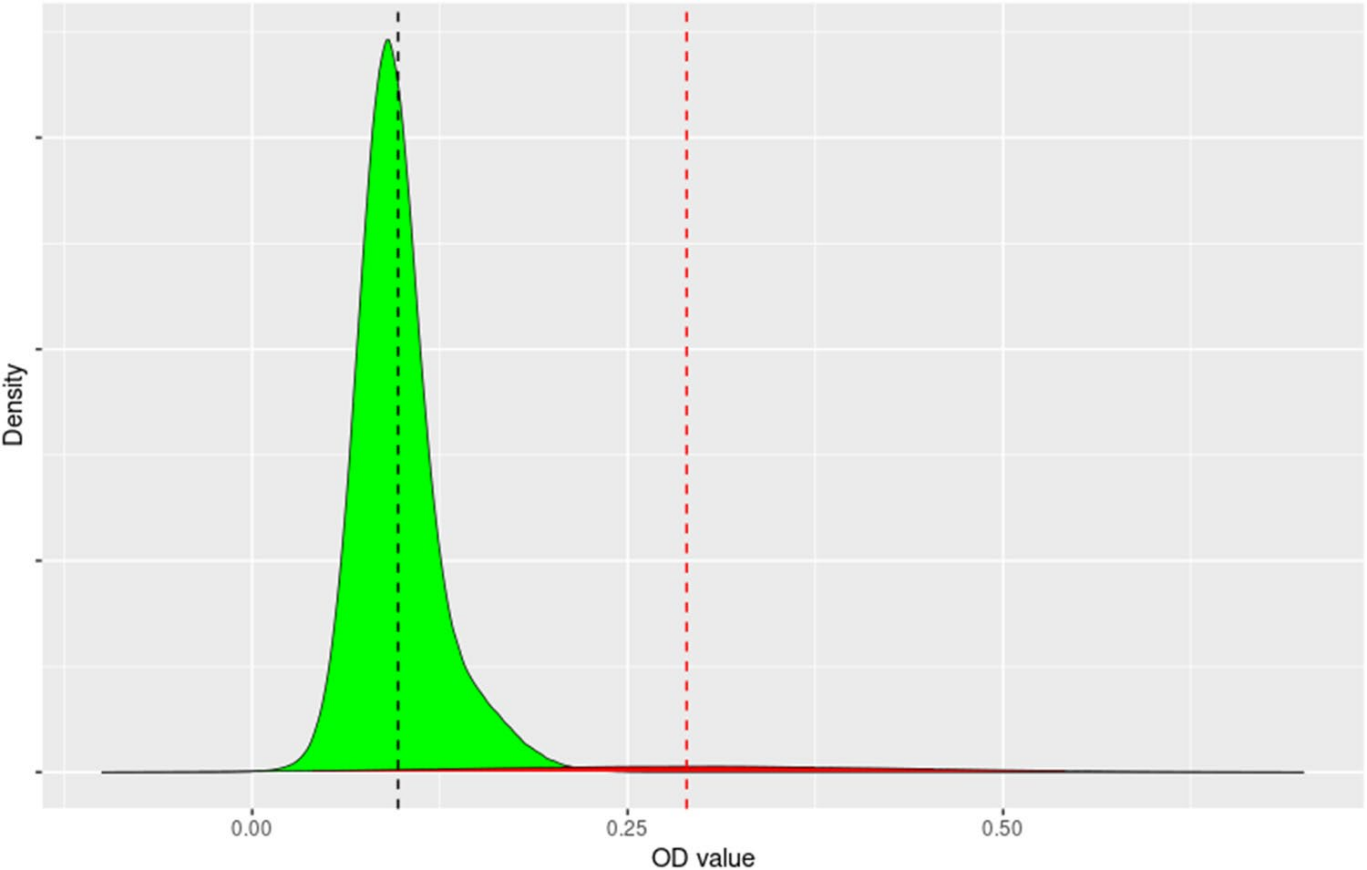
Density of ELISA OD values for negative samples (green) and positive samples (red). The mean of the negative samples (vertical dashed green line) is 0.097, whilst that of the positive samples is 0.287 (vertical red dashed line)

Analysis of the OD values using the Gaussian finite mixture models showed that the density distribution could be described by three normal distributions with a mean OD of 0.089, 0.120 and 0.287. Further analysis demonstrated that the two distributions with the lower mean ODs could be combined into a single distribution with 0.097. The presumed positive samples which were in the upper distribution with mean OD of 0.287 consisted of 2.55% of the samples, whilst 97.45% of samples were in the lower distribution and represented the presumed negative samples (Fig. 1).

**Fig. 2:**
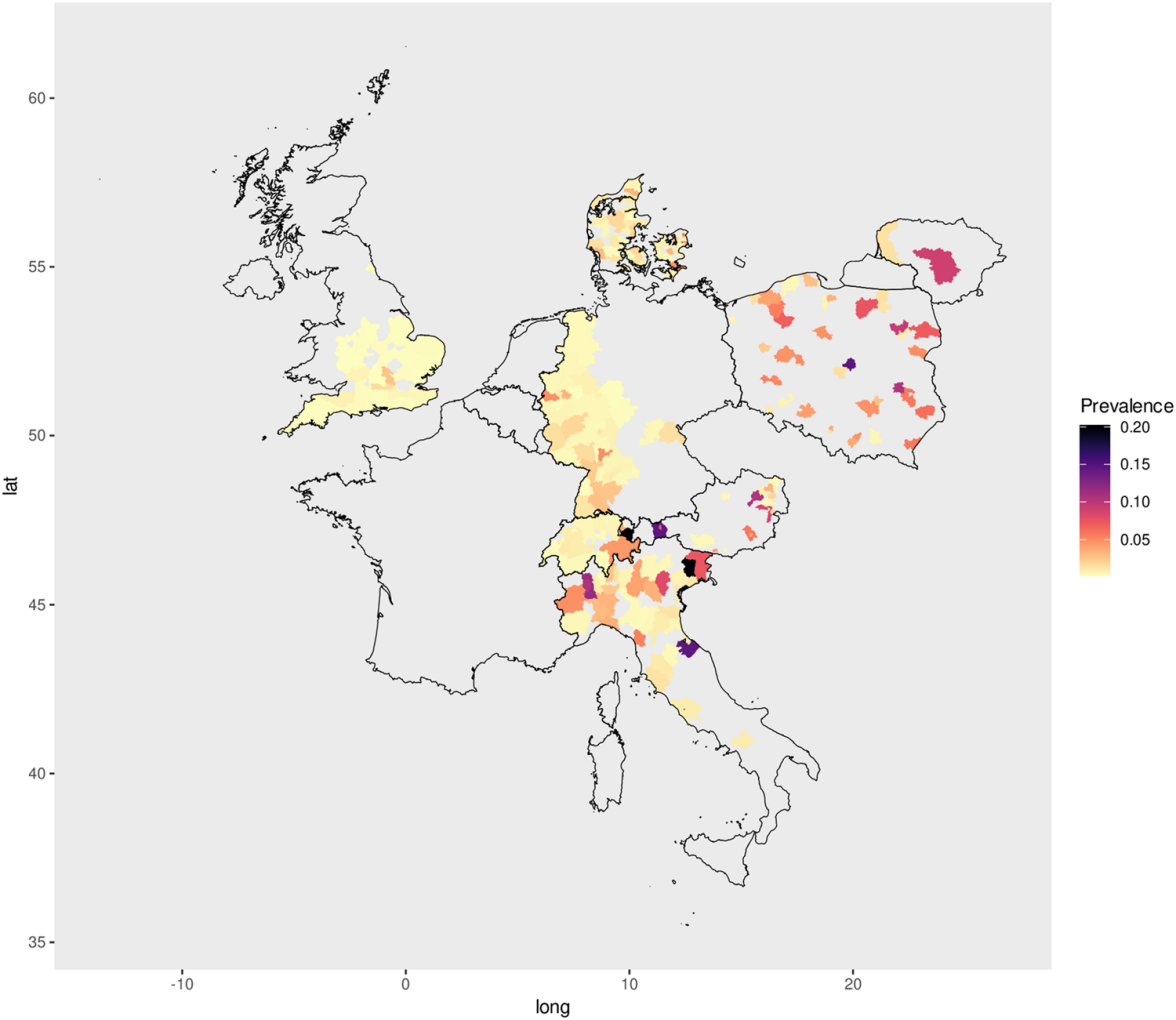
Estimated sero-prevalences for filarial infections in dogs across various European countries

The estimated prevalence of canine filariosis across various central and northern European countries is illustrated in Fig. 2.

In an animal shelter in Lithuania, the ELISA survey revealed a high prevalence, identifying it as hotspot of infection. Further investigation of blood samples from dogs at this shelter identified microfilaremia in 8 of 53 dogs. Morphometric analyses of 10 microfilariae per positive sample yielded a mean length of 362.2–388.8 µm, suggestive of *D. repens*. Furthermore, the *D. repens* diagnosis was performed and confirmed in 3 cases by PCR.

## Discussion

Identification of the European transmission areas of *D. repens* is of great importance due to its zoonotic threat. In this study, the application of a recently developed serological test (Joekel et al. 2017) based on detection of specific antibodies directed against filarial antigens allowed the estimation of the occurrence of canine filariosis based on a large number of dog sera. In contrast to southern Europe, being endemic for several filarial species, only autochthonous transmission of *D. repens* is expected in central Europe, and especially in the northern areas. This assumption was confirmed in this study by finding higher true prevalences in Poland and Lithuania, known endemic areas of *D. repens* (Alsarraf et al. 2021; Sabūnas et al. 2019), supporting also the hypothesised minor role of other filarial infections in the investigated areas*.*

The statistical approach was based on the assumption that there is no gold standard. The OD values were modelled to follow a bimodal distribution, with the upper distribution mode representing positive samples, whilst the lower distribution mode comprised the negative samples. Each individual OD value was assigned a probability of belonging to the upper modal distribution (i.e. a positive sample). The mean of these probabilities thus represents an estimate of the prevalence. The same procedure was used previously in a study on feline toxoplasmosis, where a gold standard diagnostic was not available (Schreiber et al. 2021). This approach differs from the assumption that a sample is positive if it is above a certain threshold in that no sample is classified as positive with probability 1, or vice versa. This avoids the problem of false negatives and false positives, but at the individual level there is no absolute certainty that the sample is positive or negative. However, at a population level, inferences can be made for prevalences. The mean of the probabilities that samples are positive provides an estimate of the prevalence. In our study, this allowed us to map the estimated true prevalences for seven countries in Europe.

Despite this, the method may lead to some counterintuitive results. For example, in Switzerland the estimated prevalence was 0.58, which was not dissimilar to the test prevalence of 0.77. In contrast, the UK had an estimated prevalence of 0.44 and a test prevalence of 0. This can be explained by the fact that the UK data include some false negatives based on the defined cut-off, but they belong to the positive rather than the negative model distribution (i.e. on the lower tail). The Swiss data would therefore show fewer false negatives, with the positives belonging to the upper part of the positive distribution. The high number of serum samples testing negative in the UK suggests that filarial infections in dogs remain very rare. Single positive case reports, i.e. one dog originally from Romania (Agapito et al. 2018) and one dog imported from Corfu (Wright 2017), suggest that both had a history of foreign travel.

The findings of the presented sero-prevalence of *Dirofilaria* spp. in Austria and the distribution pattern with positive foci in the eastern parts of the country are consistent with the recently reported detection of *D. repens* in military dogs in the Kaisersteinbruch region. However, the relative prevalence was only 1.4% including 94 samples with 2 positive findings (Sonnberger et al. 2021). The higher prevalence in the present study might be due to the higher number of samples examined. To the authors’ knowledge, there are no similar reports for the cluster in Western Austria. However, eggs of potential invasive mosquitoes that are competent vectors for *D. repens* transmission (*Aedes* spp.) were found along highways and in urban areas in both Eastern and Northern Tyrol (Fuehrer et al. 2020), additionally supported by autochthonous human infections (Geissler et al. 2022; Riebenbauer et al. 2021).

The distribution pattern for Germany is directed westwards in the present study, with a single cluster around the area where the federal states of Bavaria, Saxony-Anhalt and Thuringia border the Czech Republic. The first autochthonous case of *D. repens* infection was diagnosed in a southwestern region of Germany (Baden-Württemberg) (Hermosilla et al. 2006) and in three of 44 southwestern German hunting dogs that had no travel history (Pantchev et al. 2009). Additional suspected autochthonous *D. repens* infections were diagnosed in dogs from a sledge-dog kennel in Northeastern Germany, with limited travel history to Poland in winter (Sassnau et al. 2009). Furthermore, *D. repens* and *D. immitis* have been identified in mosquitoes from Southwestern and Northeastern Germany, covering the same geographic parts where autochthonous *D. repens* infections have been reported in dogs. The conclusion for this phenomenon was the suitability of the climate conditions for dirofilarial development in the mosquito vector, designating these regions at potential risk for stable endemicity (Sassnau et al. 2014a, 2014b; Sassnau and Genchi 2013). Finally, the endemicity of *D. repens* in Germany remains questionable, as most current data refer to imported or traveling pets (Pantchev et al. 2011; Schäfer et al. 2019a, b).

The test prevalence of almost 7% for Poland calculated in the present study with positive foci throughout the country confirms the results from previous studies showing that *D. repens* has become endemic in all districts of Poland (Fuehrer et al. 2021). Our finding of comparatively lower prevalence reflects the recent reported decline in the prevalence of *D. repens* in dogs in Poland (Alsarraf et al. 2021). Reasons for the decline in prevalence are thought to be increased awareness for the disease among dog owners and veterinarians, as well as preventive measures taken during the season of mosquito activity.

The low calculated test prevalence of 0.77% for Switzerland with three positively tested dog samples from Western Switzerland and one positive sample from Eastern Switzerland does not allow a statement about the origin of occurrence of *D. repens* within the country. Based on these findings, neither an autochthonous occurrence of the parasite nor imported cases could be confirmed. Moreover, most of the cases of dirofilariosis reported to date have a confirmed history of import or residence abroad (Fuehrer et al. 2021; Glaus et al. 2019), including positive dogs from southern Switzerland (Ticino), considered to be the border of the endemic area for both *Dirofilaria* spp. (Fuehrer et al. 2021).

To the authors’ knowledge, there are no published reports of *Dirofilaria* spp. in animals in Denmark. Our findings of 28 positive samples out of 1440 remain new. However, the measured OD value was only slightly above the cut-off value and no further tests were performed to confirm positivity. Therefore, false-positive test results are still possible.

Compared to previously published data on 8.3% prevalence percentages (25 positives out of 300 dog sera samples) in the Abruzzo region of central Italy (Traversa et al. 2010), the present test prevalence of 3.37% appears low. For *D. immitis* infections, data from more than 10′000 serological assays performed between 2009 and 2019 identified changing patterns between northern, central and southern Italy, with an overall gradual increase over time (Mendoza-Roldan et al. 2020). Recent studies and a questionnaire study showed that clinical infections with *D. immitis* and *D. repens* remain frequent diagnoses in veterinary clinics in the country (Ferrara et al. 2022Genchi et al. 2019; Macchioni et al. 2020).

Our comprehensive investigation of samples from Lithuania (ELISA, Knott, morphometric measurements and PCR) clearly confirms the endemic occurrence of *D. repens* in the Kaunas region of Central Lithuania. The samples were from dogs that lived in animal shelters that had not travelled before. Previously reported positive cases of *D. repens* in dogs (61 positives from 2280 blood samples) and seven confirmed cases of human infections support these findings. Moreover, a significantly higher infection rate was found in dogs from animal shelters than in pet dogs (Sabūnas et al. 2019).

Overall, the present study represents a novel approach to illustrate the putative occurence of filarial infections in Central Europe by combining ELISA with the statistical approach using OD density curves, and finally mapping the mean probabilities on a sample level.

## Data Availability

Data are available on request from the authors.
